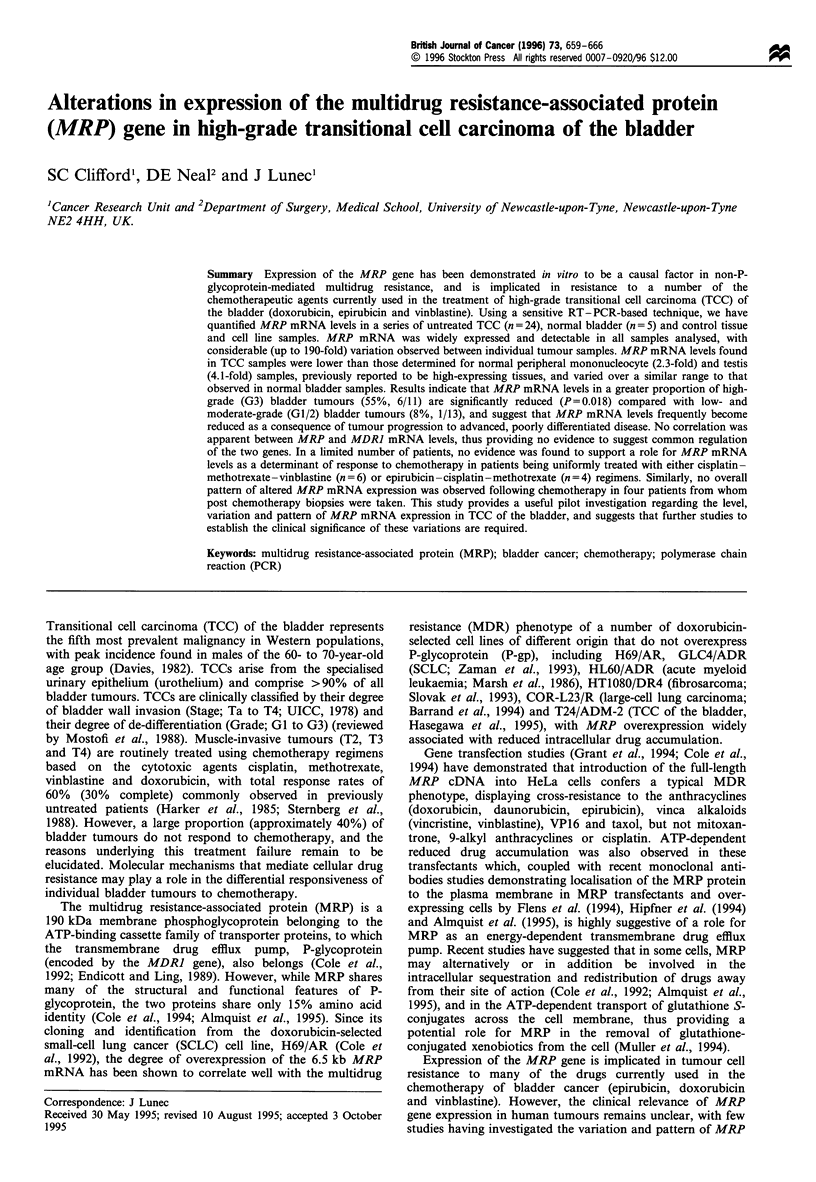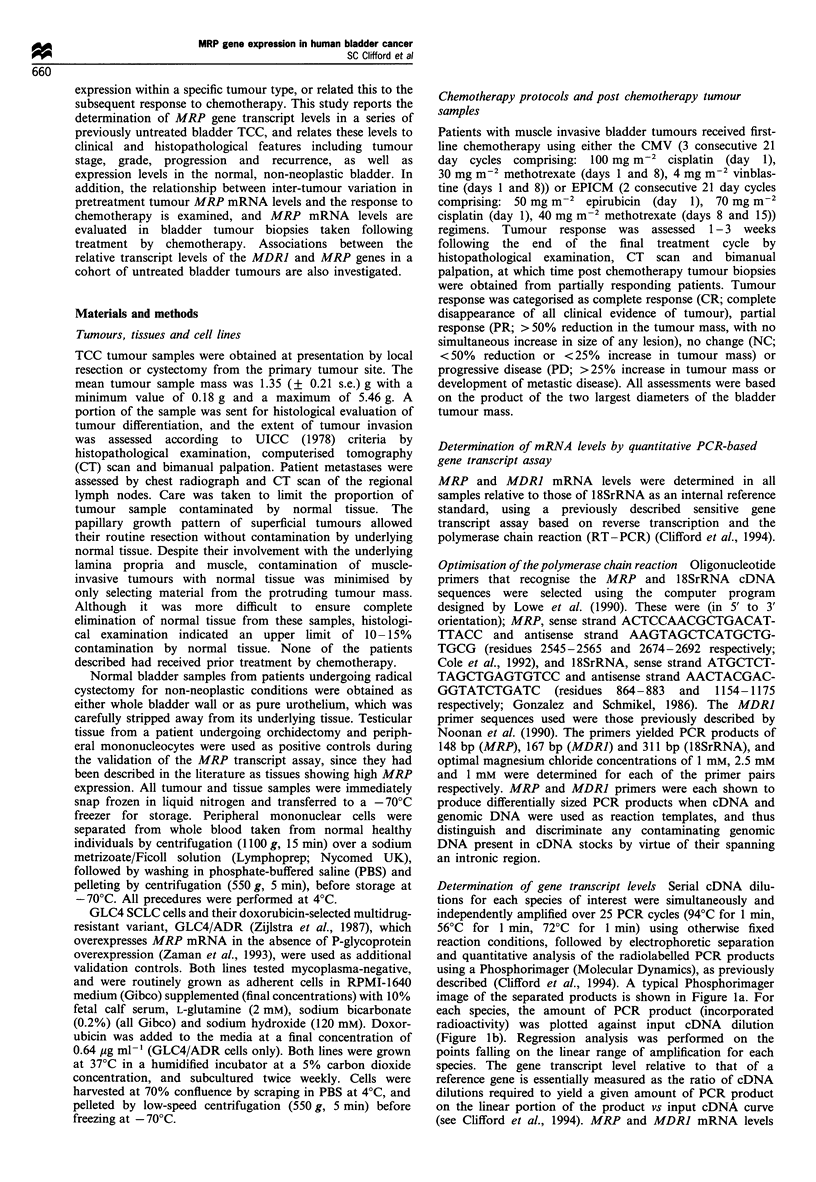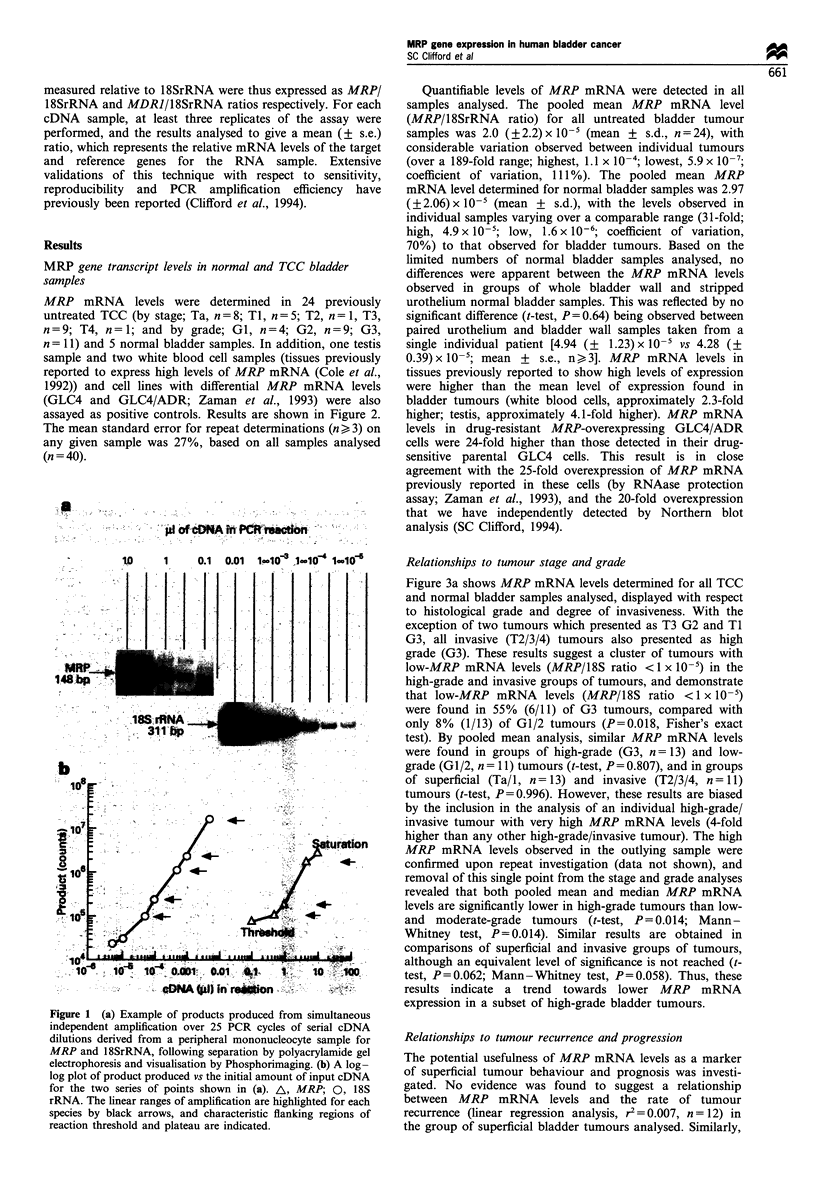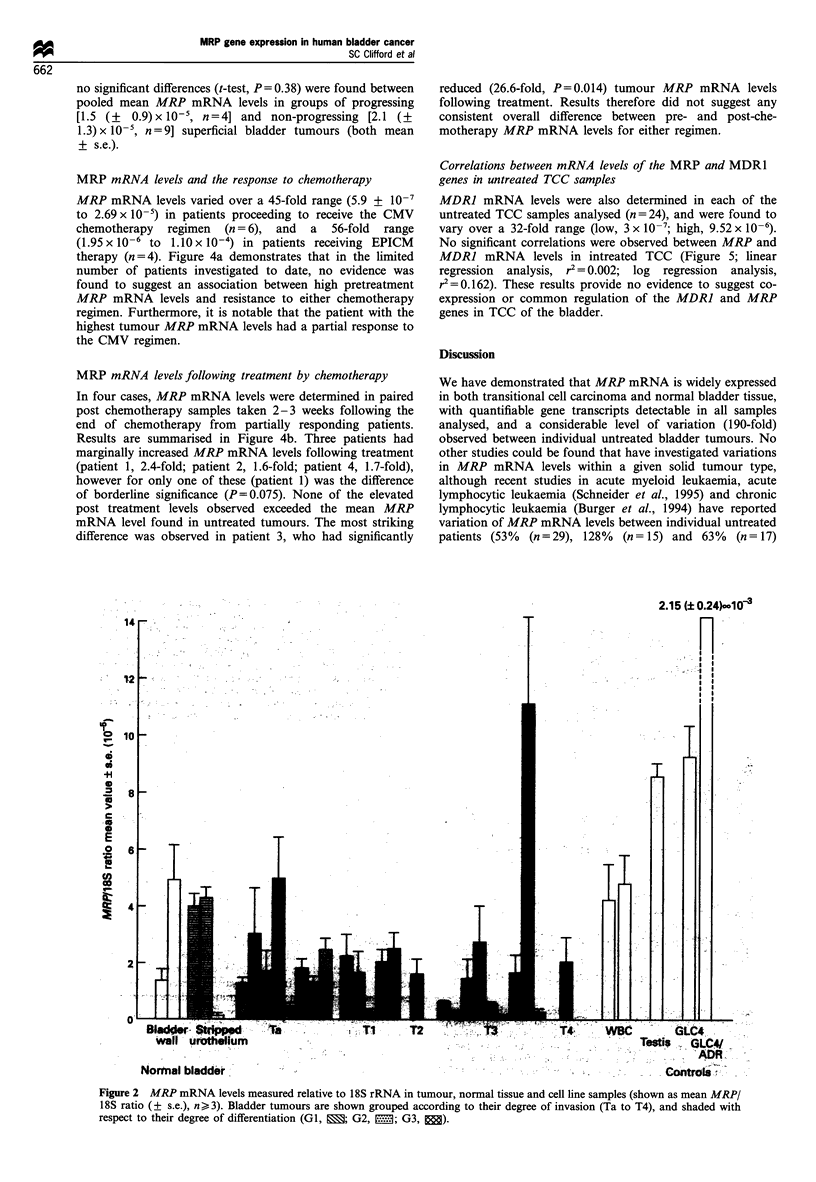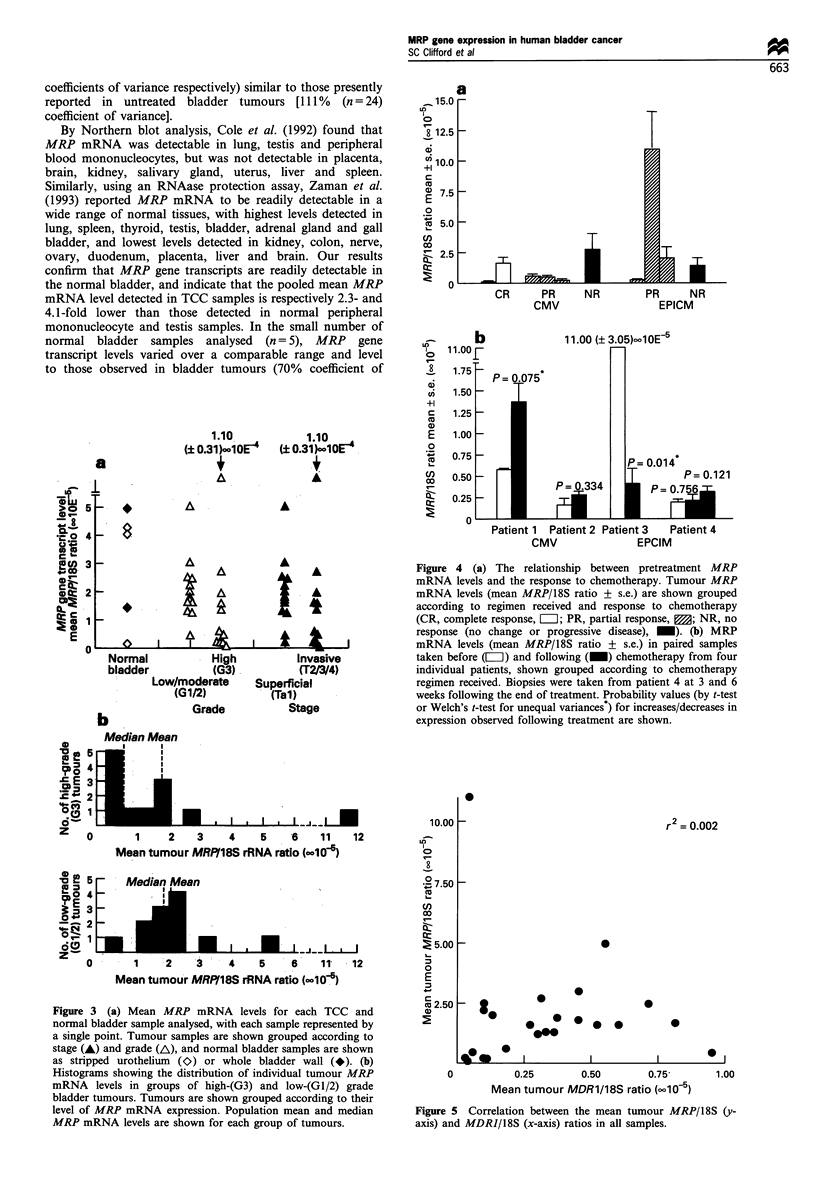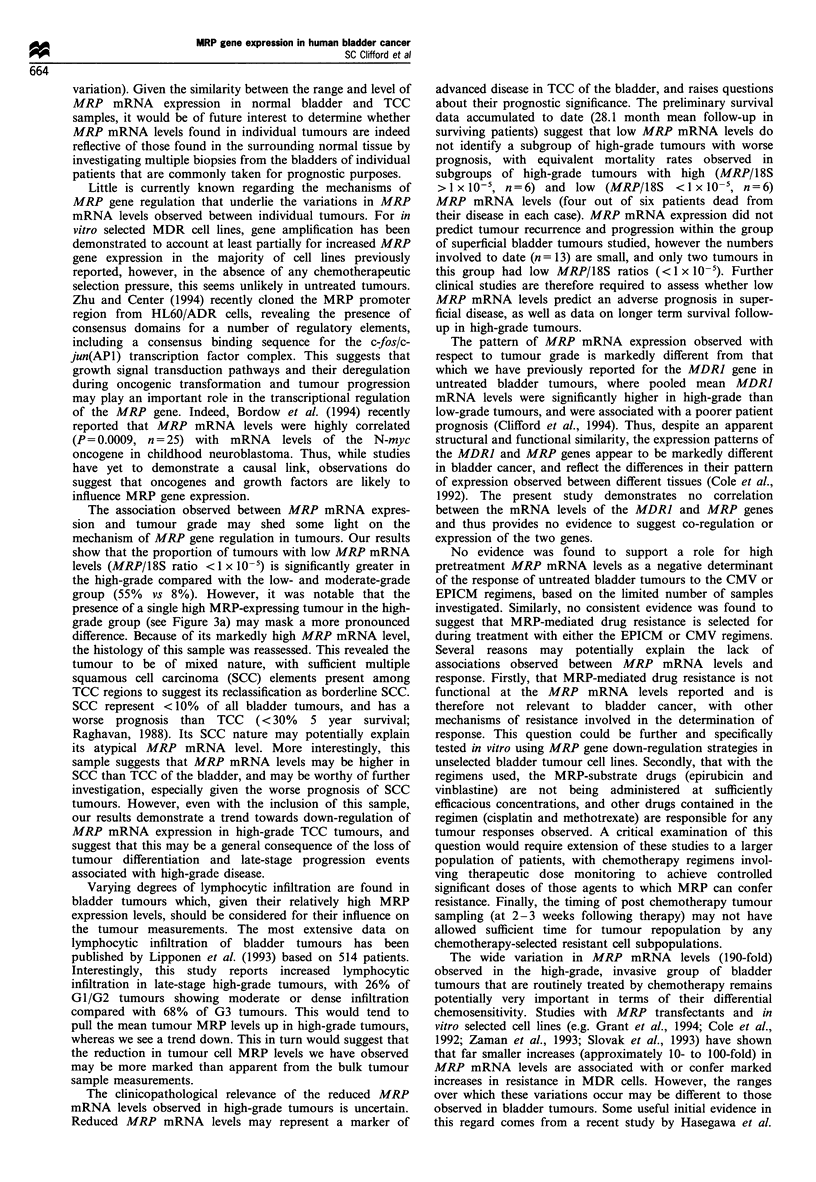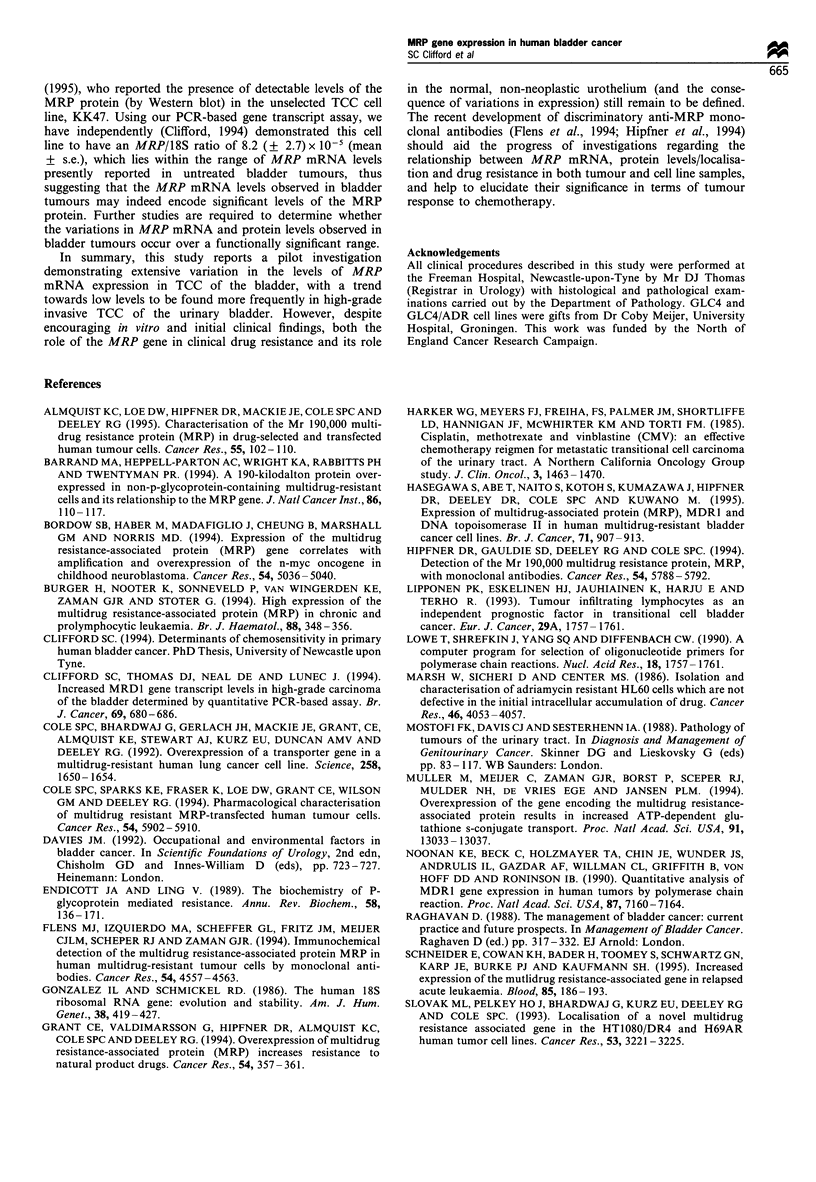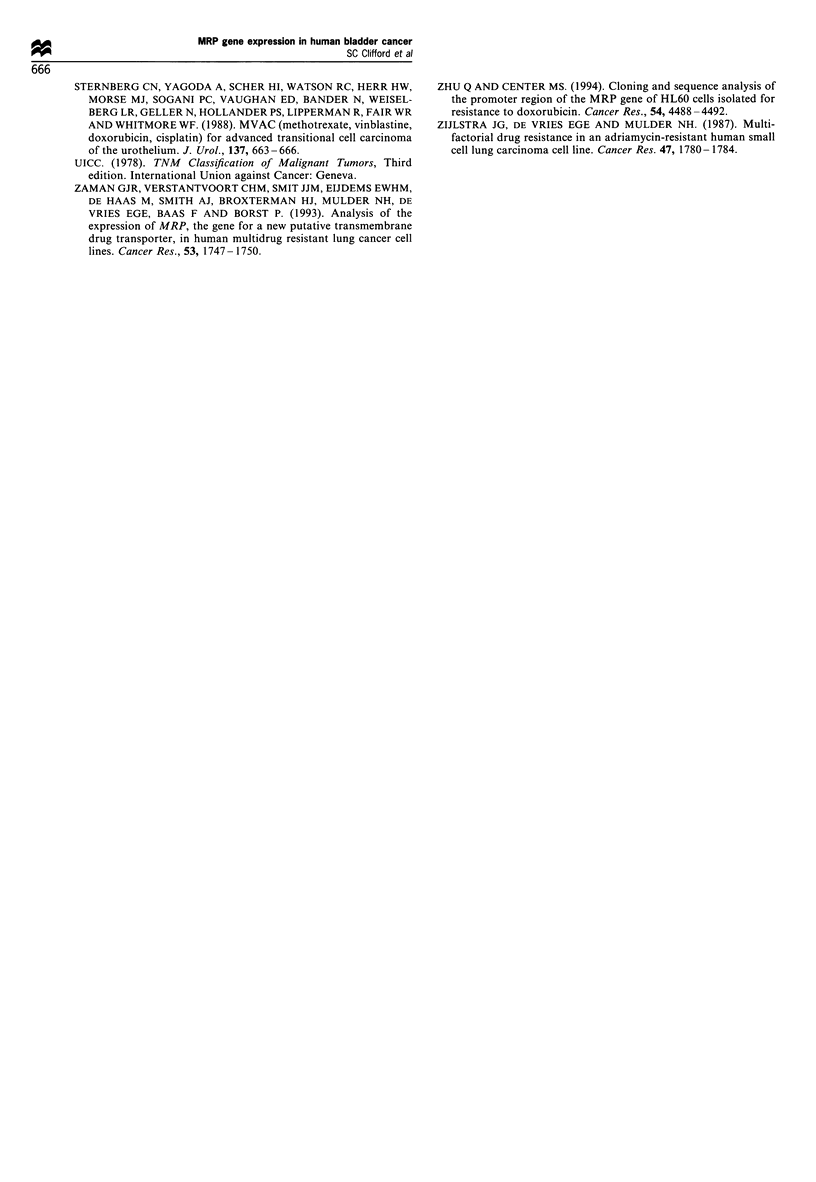# Alterations in expression of the multidrug resistance-associated protein (MRP) gene in high-grade transitional cell carcinoma of the bladder.

**DOI:** 10.1038/bjc.1996.115

**Published:** 1996-03

**Authors:** S. C. Clifford, D. E. Neal, J. Lunec

**Affiliations:** Cancer Research Unit, Medical School, University of Newcastle-upon-Tyne, UK.

## Abstract

**Images:**


British Journal of Cancer (1996) 73, 659-666
© 1996 Stockton Press All rights reserved 0007-0920/96 $12.00

### Alterations in expression of the multidrug resistance-associated protein (*MRP*) gene in high-grade transitional cell carcinoma of the bladder

SC Clifford[1], DE Neal[2] and J Lunec[1]

[1]*Cancer Research Unit and* [2]*Department of Surgery, Medical School, University of Newcastle-upon-Tyne, Newcastle-upon-Tyne NE2 4HH, UK.*

**Summary** Expression of the *MRP* gene has been demonstrated *in vitro* to be a causal factor in non-P-glycoprotein-mediated multidrug resistance, and is implicated in resistance to a number of the chemotherapeutic agents currently used in the treatment of high-grade transitional cell carcinoma (TCC) of the bladder (doxorubicin, epirubicin and vinblastine). Using a sensitive RT-PCR-based technique, we have quantified *MRP* mRNA levels in a series of untreated TCC ($n=24$), normal bladder ($n=5$) and control tissue and cell line samples. *MRP* mRNA was widely expressed and detectable in all samples analysed, with considerable (up to 190-fold) variation observed between individual tumour samples. *MRP* mRNA levels found in TCC samples were lower than those determined for normal peripheral mononucleocyte (2.3-fold) and testis (4.1-fold) samples, previously reported to be high-expressing tissues, and varied over a similar range to that observed in normal bladder samples. Results indicate that *MRP* mRNA levels in a greater proportion of high-grade (G3) bladder tumours (55%, 6/11) are significantly reduced ($P=0.018$) compared with low- and moderate-grade (G1/2) bladder tumours (8%, 1/13), and suggest that *MRP* mRNA levels frequently become reduced as a consequence of tumour progression to advanced, poorly differentiated disease. No correlation was apparent between *MRP* and *MDR1* mRNA levels, thus providing no evidence to suggest common regulation of the two genes. In a limited number of patients, no evidence was found to support a role for *MRP* mRNA levels as a determinant of response to chemotherapy in patients being uniformly treated with either cisplatin-methotrexate-vinblastine ($n=6$) or epirubicin-cisplatin-methotrexate ($n=4$) regimens. Similarly, no overall pattern of altered *MRP* mRNA expression was observed following chemotherapy in four patients from whom post chemotherapy biopsies were taken. This study provides a useful pilot investigation regarding the level, variation and pattern of *MRP* mRNA expression in TCC of the bladder, and suggests that further studies to establish the clinical significance of these variations are required.

**Keywords:** multidrug resistance-associated protein (MRP); bladder cancer; chemotherapy; polymerase chain reaction (PCR)

Transitional cell carcinoma (TCC) of the bladder represents the fifth most prevalent malignancy in Western populations, with peak incidence found in males of the 60- to 70-year-old age group (Davies, 1982). TCCs arise from the specialised urinary epithelium (urothelium) and comprise >90% of all bladder tumours. TCCs are clinically classified by their degree of bladder wall invasion (Stage; Ta to T4; UICC, 1978) and their degree of de-differentiation (Grade; G1 to G3) (reviewed by Mostofi *et al.*, 1988). Muscle-invasive tumours (T2, T3 and T4) are routinely treated using chemotherapy regimens based on the cytotoxic agents cisplatin, methotrexate, vinblastine and doxorubicin, with total response rates of 60% (30% complete) commonly observed in previously untreated patients (Harker *et al.*, 1985; Sternberg *et al.*, 1988). However, a large proportion (approximately 40%) of bladder tumours do not respond to chemotherapy, and the reasons underlying this treatment failure remain to be elucidated. Molecular mechanisms that mediate cellular drug resistance may play a role in the differential responsiveness of individual bladder tumours to chemotherapy.

The multidrug resistance-associated protein (MRP) is a 190 kDa membrane phosphoglycoprotein belonging to the ATP-binding cassette family of transporter proteins, to which the transmembrane drug efflux pump, P-glycoprotein (encoded by the *MDR1* gene), also belongs (Cole *et al.*, 1992; Endicott and Ling, 1989). However, while MRP shares many of the structural and functional features of P-glycoprotein, the two proteins share only 15% amino acid identity (Cole *et al.*, 1994; Almquist *et al.*, 1995). Since its cloning and identification from the doxorubicin-selected small-cell lung cancer (SCLC) cell line, H69/AR (Cole *et al.*, 1992), the degree of overexpression of the 6.5 kb *MRP* mRNA has been shown to correlate well with the multidrug resistance (MDR) phenotype of a number of doxorubicin-selected cell lines of different origin that do not overexpress P-glycoprotein (P-gp), including H69/AR, GLC4/ADR (SCLC; Zaman *et al.*, 1993), HL60/ADR (acute myeloid leukaemia; Marsh *et al.*, 1986), HT1080/DR4 (fibrosarcoma; Slovak *et al.*, 1993), COR-L23/R (large-cell lung carcinoma; Barrand *et al.*, 1994) and T24/ADM-2 (TCC of the bladder, Hasegawa *et al.*, 1995), with *MRP* overexpression widely associated with reduced intracellular drug accumulation.

Gene transfection studies (Grant *et al.*, 1994; Cole *et al.*, 1994) have demonstrated that introduction of the full-length *MRP* cDNA into HeLa cells confers a typical MDR phenotype, displaying cross-resistance to the anthracyclines (doxorubicin, daunorubicin, epirubicin), vinca alkaloids (vincristine, vinblastine), VP16 and taxol, but not mitoxantrone, 9-alkyl anthracyclines or cisplatin. ATP-dependent reduced drug accumulation was also observed in these transfectants which, coupled with recent monoclonal antibodies studies demonstrating localisation of the MRP protein to the plasma membrane in MRP transfectants and overexpressing cells by Flens *et al.* (1994), Hipfner *et al.* (1994) and Almquist *et al.* (1995), is highly suggestive of a role for MRP as an energy-dependent transmembrane drug efflux pump. Recent studies have suggested that in some cells, MRP may alternatively or in addition be involved in the intracellular sequestration and redistribution of drugs away from their site of action (Cole *et al.*, 1992; Almquist *et al.*, 1995), and in the ATP-dependent transport of glutathione *S*-conjugates across the cell membrane, thus providing a potential role for MRP in the removal of glutathione-conjugated xenobiotics from the cell (Muller *et al.*, 1994).

Expression of the *MRP* gene is implicated in tumour cell resistance to many of the drugs currently used in the chemotherapy of bladder cancer (epirubicin, doxorubicin and vinblastine). However, the clinical relevance of *MRP* gene expression in human tumours remains unclear, with few studies having investigated the variation and pattern of *MRP*

Correspondence: J Lunec
Received 30 May 1995; revised 10 August 1995; accepted 3 October 1995

expression within a specific tumour type, or related this to the subsequent response to chemotherapy. This study reports the determination of *MRP* gene transcript levels in a series of previously untreated bladder TCC, and relates these levels to clinical and histopathological features including tumour stage, grade, progression and recurrence, as well as expression levels in the normal, non-neoplastic bladder. In addition, the relationship between inter-tumour variation in pretreatment tumour *MRP* mRNA levels and the response to chemotherapy is examined, and *MRP* mRNA levels are evaluated in bladder tumour biopsies taken following treatment by chemotherapy. Associations between the relative transcript levels of the *MDR1* and *MRP* genes in a cohort of untreated bladder tumours are also investigated.

### Materials and methods

### *Tumours, tissues and cell lines*

TCC tumour samples were obtained at presentation by local resection or cystectomy from the primary tumour site. The mean tumour sample mass was 1.35 ($\pm$ 0.21 s.e.) g with a minimum value of 0.18 g and a maximum of 5.46 g. A portion of the sample was sent for histological evaluation of tumour differentiation, and the extent of tumour invasion was assessed according to UICC (1978) criteria by histopathological examination, computerised tomography (CT) scan and bimanual palpation. Patient metastases were assessed by chest radiograph and CT scan of the regional lymph nodes. Care was taken to limit the proportion of tumour sample contaminated by normal tissue. The papillary growth pattern of superficial tumours allowed their routine resection without contamination by underlying normal tissue. Despite their involvement with the underlying lamina propria and muscle, contamination of muscle-invasive tumours with normal tissue was minimised by only selecting material from the protruding tumour mass. Although it was more difficult to ensure complete elimination of normal tissue from these samples, histological examination indicated an upper limit of 10–15% contamination by normal tissue. None of the patients described had received prior treatment by chemotherapy.

Normal bladder samples from patients undergoing radical cystectomy for non-neoplastic conditions were obtained as either whole bladder wall or as pure urothelium, which was carefully stripped away from its underlying tissue. Testicular tissue from a patient undergoing orchidectomy and peripheral mononucleocytes were used as positive controls during the validation of the *MRP* transcript assay, since they had been described in the literature as tissues showing high *MRP* expression. All tumour and tissue samples were immediately snap frozen in liquid nitrogen and transferred to a −70°C freezer for storage. Peripheral mononuclear cells were separated from whole blood taken from normal healthy individuals by centrifugation (1100 *g*, 15 min) over a sodium metrizoate/Ficoll solution (Lymphoprep; Nycomed UK), followed by washing in phosphate-buffered saline (PBS) and pelleting by centrifugation (550 *g*, 5 min), before storage at −70°C. All precedures were performed at 4°C.

GLC4 SCLC cells and their doxorubicin-selected multidrug-resistant variant, GLC4/ADR (Zijlstra *et al*., 1987), which overexpresses *MRP* mRNA in the absence of P-glycoprotein overexpression (Zaman *et al*., 1993), were used as additional validation controls. Both lines tested mycoplasma-negative, and were routinely grown as adherent cells in RPMI-1640 medium (Gibco) supplemented (final concentrations) with 10% fetal calf serum, L-glutamine (2 mM), sodium bicarbonate (0.2%) (all Gibco) and sodium hydroxide (120 mM). Doxorubicin was added to the media at a final concentration of 0.64 $\mu$g $ml^{-1}$ (GLC4/ADR cells only). Both lines were grown at 37°C in a humidified incubator at a 5% carbon dioxide concentration, and subcultured twice weekly. Cells were harvested at 70% confluence by scraping in PBS at 4°C, and pelleted by low-speed centrifugation (550 *g*, 5 min) before freezing at −70°C.

### *Chemotherapy protocols and post chemotherapy tumour samples*

Patients with muscle invasive bladder tumours received first-line chemotherapy using either the CMV (3 consecutive 21 day cycles comprising: 100 mg $m^{-2}$ cisplatin (day 1), 30 mg $m^{-2}$ methotrexate (days 1 and 8), 4 mg $m^{-2}$ vinblastine (days 1 and 8)) or EPICM (2 consecutive 21 day cycles comprising: 50 mg $m^{-2}$ epirubicin (day 1), 70 mg $m^{-2}$ cisplatin (day 1), 40 mg $m^{-2}$ methotrexate (days 8 and 15)) regimens. Tumour response was assessed 1–3 weeks following the end of the final treatment cycle by histopathological examination, CT scan and bimanual palpation, at which time post chemotherapy tumour biopsies were obtained from partially responding patients. Tumour response was categorised as complete response (CR; complete disappearance of all clinical evidence of tumour), partial response (PR; >50% reduction in the tumour mass, with no simultaneous increase in size of any lesion), no change (NC; <50% reduction or <25% increase in tumour mass) or progressive disease (PD; >25% increase in tumour mass or development of metastic disease). All assessments were based on the product of the two largest diameters of the bladder tumour mass.

### *Determination of mRNA levels by quantitative PCR-based gene transcript assay*

*MRP* and *MDR1* mRNA levels were determined in all samples relative to those of 18SrRNA as an internal reference standard, using a previously described sensitive gene transcript assay based on reverse transcription and the polymerase chain reaction (RT–PCR) (Clifford *et al*., 1994).

*Optimisation of the polymerase chain reaction* Oligonucleotide primers that recognise the *MRP* and 18SrRNA cDNA sequences were selected using the computer program designed by Lowe *et al*. (1990). These were (in 5′ to 3′ orientation); *MRP*, sense strand ACTCCAACGCTGACAT-TTACC and antisense strand AAGTAGCTCATGCTG-TGCG (residues 2545–2565 and 2674–2692 respectively; Cole *et al*., 1992), and 18SrRNA, sense strand ATGCTCT-TAGCTGAGTGTCC and antisense strand AACTACGAC-GGTATCTGATC (residues 864–883 and 1154–1175 respectively; Gonzalez and Schmikel, 1986). The *MDR1* primer sequences used were those previously described by Noonan *et al*. (1990). The primers yielded PCR products of 148 bp (*MRP*), 167 bp (*MDR1*) and 311 bp (18SrRNA), and optimal magnesium chloride concentrations of 1 mM, 2.5 mM and 1 mM were determined for each of the primer pairs respectively. *MRP* and *MDR1* primers were each shown to produce differentially sized PCR products when cDNA and genomic DNA were used as reaction templates, and thus distinguish and discriminate any contaminating genomic DNA present in cDNA stocks by virtue of their spanning an intronic region.

*Determination of gene transcript levels* Serial cDNA dilutions for each species of interest were simultaneously and independently amplified over 25 PCR cycles (94°C for 1 min, 56°C for 1 min, 72°C for 1 min) using otherwise fixed reaction conditions, followed by electrophoretic separation and quantitative analysis of the radiolabelled PCR products using a Phosphorimager (Molecular Dynamics), as previously described (Clifford *et al*., 1994). A typical Phosphorimager image of the separated products is shown in Figure 1a. For each species, the amount of PCR product (incorporated radioactivity) was plotted against input cDNA dilution (Figure 1b). Regression analysis was performed on the points falling on the linear range of amplification for each species. The gene transcript level relative to that of a reference gene is essentially measured as the ratio of cDNA dilutions required to yield a given amount of PCR product on the linear portion of the product *vs* input cDNA curve (see Clifford *et al*., 1994). *MRP* and *MDR1* mRNA levels measured relative to 18SrRNA were thus expressed as *MRP*/18SrRNA and *MDR1*/18SrRNA ratios respectively. For each cDNA sample, at least three replicates of the assay were performed, and the results analysed to give a mean (± s.e.) ratio, which represents the relative mRNA levels of the target and reference genes for the RNA sample. Extensive validations of this technique with respect to sensitivity, reproducibility and PCR amplification efficiency have previously been reported (Clifford *et al*., 1994).

### Results

### MRP *gene transcript levels in normal and TCC bladder samples*

*MRP* mRNA levels were determined in 24 previously untreated TCC (by stage; Ta, $n=8$; T1, $n=5$; T2, $n=1$, T3, $n=9$; T4, $n=1$; and by grade; G1, $n=4$; G2, $n=9$; G3, $n=11$) and 5 normal bladder samples. In addition, one testis sample and two white blood cell samples (tissues previously reported to express high levels of *MRP* mRNA (Cole *et al*., 1992)) and cell lines with differential *MRP* mRNA levels (GLC4 and GLC4/ADR; Zaman *et al*., 1993) were also assayed as positive controls. Results are shown in Figure 2. The mean standard error for repeat determinations ($n \geq 3$) on any given sample was 27%, based on all samples analysed ($n=40$).

**Figure 1** (**a**) Example of products produced from simultaneous independent amplification over 25 PCR cycles of serial cDNA dilutions derived from a peripheral mononucleocyte sample for *MRP* and 18SrRNA, following separation by polyacrylamide gel electrophoresis and visualisation by Phosphorimaging. (**b**) A log–log plot of product produced *vs* the initial amount of input cDNA for the two series of points shown in (**a**). △, *MRP*; ○, 18S rRNA. The linear ranges of amplification are highlighted for each species by black arrows, and characteristic flanking regions of reaction threshold and plateau are indicated.

Quantifiable levels of *MRP* mRNA were detected in all samples analysed. The pooled mean *MRP* mRNA level (*MRP*/18SrRNA ratio) for all untreated bladder tumour samples was 2.0 $(\pm 2.2) \times 10^{-5}$ (mean ± s.d., $n=24$), with considerable variation observed between individual tumours (over a 189-fold range; highest, $1.1 \times 10^{-4}$; lowest, $5.9 \times 10^{-7}$; coefficient of variation, 111%). The pooled mean *MRP* mRNA level determined for normal bladder samples was 2.97 $(\pm 2.06) \times 10^{-5}$ (mean ± s.d.), with the levels observed in individual samples varying over a comparable range (31-fold; high, $4.9 \times 10^{-5}$; low, $1.6 \times 10^{-6}$; coefficient of variation, 70%) to that observed for bladder tumours. Based on the limited numbers of normal bladder samples analysed, no differences were apparent between the *MRP* mRNA levels observed in groups of whole bladder wall and stripped urothelium normal bladder samples. This was reflected by no significant difference (*t*-test, $P=0.64$) being observed between paired urothelium and bladder wall samples taken from a single individual patient [4.94 $(\pm\ 1.23) \times 10^{-5}$ *vs* 4.28 $(\pm\ 0.39) \times 10^{-5}$; mean ± s.e., $n \geq 3$]. *MRP* mRNA levels in tissues previously reported to show high levels of expression were higher than the mean level of expression found in bladder tumours (white blood cells, approximately 2.3-fold higher; testis, approximately 4.1-fold higher). *MRP* mRNA levels in drug-resistant *MRP*-overexpressing GLC4/ADR cells were 24-fold higher than those detected in their drug-sensitive parental GLC4 cells. This result is in close agreement with the 25-fold overexpression of *MRP* mRNA previously reported in these cells (by RNAase protection assay; Zaman *et al*., 1993), and the 20-fold overexpression that we have independently detected by Northern blot analysis (SC Clifford, 1994).

### *Relationships to tumour stage and grade*

Figure 3a shows *MRP* mRNA levels determined for all TCC and normal bladder samples analysed, displayed with respect to histological grade and degree of invasiveness. With the exception of two tumours which presented as T3 G2 and T1 G3, all invasive (T2/3/4) tumours also presented as high grade (G3). These results suggest a cluster of tumours with low-*MRP* mRNA levels (*MRP*/18S ratio $<1 \times 10^{-5}$) in the high-grade and invasive groups of tumours, and demonstrate that low-*MRP* mRNA levels (*MRP*/18S ratio $<1 \times 10^{-5}$) were found in 55% (6/11) of G3 tumours, compared with only 8% (1/13) of G1/2 tumours ($P=0.018$, Fisher's exact test). By pooled mean analysis, similar *MRP* mRNA levels were found in groups of high-grade (G3, $n=13$) and low-grade (G1/2, $n=11$) tumours (*t*-test, $P=0.807$), and in groups of superficial (Ta/1, $n=13$) and invasive (T2/3/4, $n=11$) tumours (*t*-test, $P=0.996$). However, these results are biased by the inclusion in the analysis of an individual high-grade/invasive tumour with very high *MRP* mRNA levels (4-fold higher than any other high-grade/invasive tumour). The high *MRP* mRNA levels observed in the outlying sample were confirmed upon repeat investigation (data not shown), and removal of this single point from the stage and grade analyses revealed that both pooled mean and median *MRP* mRNA levels are significantly lower in high-grade tumours than low- and moderate-grade tumours (*t*-test, $P=0.014$; Mann–Whitney test, $P=0.014$). Similar results are obtained in comparisons of superficial and invasive groups of tumours, although an equivalent level of significance is not reached (*t*-test, $P=0.062$; Mann–Whitney test, $P=0.058$). Thus, these results indicate a trend towards lower *MRP* mRNA expression in a subset of high-grade bladder tumours.

### *Relationships to tumour recurrence and progression*

The potential usefulness of *MRP* mRNA levels as a marker of superficial tumour behaviour and prognosis was investigated. No evidence was found to suggest a relationship between *MRP* mRNA levels and the rate of tumour recurrence (linear regression analysis, $r^2=0.007$, $n=12$) in the group of superficial bladder tumours analysed. Similarly, no significant differences ($t$-test, $P=0.38$) were found between pooled mean *MRP* mRNA levels in groups of progressing [1.5 ($\pm$ 0.9)$\times 10^{-5}$, $n=4$] and non-progressing [2.1 ($\pm$ 1.3)$\times 10^{-5}$, $n=9$] superficial bladder tumours (both mean $\pm$ s.e.).

### MRP *mRNA levels and the response to chemotherapy*

*MRP* mRNA levels varied over a 45-fold range (5.9 $\pm$ $10^{-7}$ to $2.69\times 10^{-5}$) in patients proceeding to receive the CMV chemotherapy regimen ($n=6$), and a 56-fold range ($1.95\times 10^{-6}$ to $1.10\times 10^{-4}$) in patients receiving EPICM therapy ($n=4$). Figure 4a demonstrates that in the limited number of patients investigated to date, no evidence was found to suggest an association between high pretreatment *MRP* mRNA levels and resistance to either chemotherapy regimen. Furthermore, it is notable that the patient with the highest tumour *MRP* mRNA levels had a partial response to the CMV regimen.

### MRP *mRNA levels following treatment by chemotherapy*

In four cases, *MRP* mRNA levels were determined in paired post chemotherapy samples taken 2–3 weeks following the end of chemotherapy from partially responding patients. Results are summarised in Figure 4b. Three patients had marginally increased *MRP* mRNA levels following treatment (patient 1, 2.4-fold; patient 2, 1.6-fold; patient 4, 1.7-fold), however for only one of these (patient 1) was the difference of borderline significance ($P=0.075$). None of the elevated post treatment levels observed exceeded the mean *MRP* mRNA level found in untreated tumours. The most striking difference was observed in patient 3, who had significantly reduced (26.6-fold, $P=0.014$) tumour *MRP* mRNA levels following treatment. Results therefore did not suggest any consistent overall difference between pre- and post-chemotherapy *MRP* mRNA levels for either regimen.

### *Correlations between mRNA levels of the* MRP *and* MDR1 *genes in untreated TCC samples*

*MDR1* mRNA levels were also determined in each of the untreated TCC samples analysed ($n=24$), and were found to vary over a 32-fold range (low, $3\times 10^{-7}$; high, $9.52\times 10^{-6}$). No significant correlations were observed between *MRP* and *MDR1* mRNA levels in intreated TCC (Figure 5; linear regression analysis, $r^2=0.002$; log regression analysis, $r^2=0.162$). These results provide no evidence to suggest co-expression or common regulation of the *MDR1* and *MRP* genes in TCC of the bladder.

### Discussion

We have demonstrated that *MRP* mRNA is widely expressed in both transitional cell carcinoma and normal bladder tissue, with quantifiable gene transcripts detectable in all samples analysed, and a considerable level of variation (190-fold) observed between individual untreated bladder tumours. No other studies could be found that have investigated variations in *MRP* mRNA levels within a given solid tumour type, although recent studies in acute myeloid leukaemia, acute lymphocytic leukaemia (Schneider *et al.*, 1995) and chronic lymphocytic leukaemia (Burger *et al.*, 1994) have reported variation of *MRP* mRNA levels between individual untreated patients (53% ($n=29$), 128% ($n=15$) and 63% ($n=17$)

**Figure 2** *MRP* mRNA levels measured relative to 18S rRNA in tumour, normal tissue and cell line samples (shown as mean *MRP*/18S ratio ($\pm$ s.e.), $n\geqslant 3$). Bladder tumours are shown grouped according to their degree of invasion (Ta to T4), and shaded with respect to their degree of differentiation (G1, ; G2, ; G3, ).

coefficients of variance respectively) similar to those presently reported in untreated bladder tumours [111% ($n=24$) coefficient of variance].

By Northern blot analysis, Cole *et al.* (1992) found that *MRP* mRNA was detectable in lung, testis and peripheral blood mononucleocytes, but was not detectable in placenta, brain, kidney, salivary gland, uterus, liver and spleen. Similarly, using an RNAase protection assay, Zaman *et al.* (1993) reported *MRP* mRNA to be readily detectable in a wide range of normal tissues, with highest levels detected in lung, spleen, thyroid, testis, bladder, adrenal gland and gall bladder, and lowest levels detected in kidney, colon, nerve, ovary, duodenum, placenta, liver and brain. Our results confirm that *MRP* gene transcripts are readily detectable in the normal bladder, and indicate that the pooled mean *MRP* mRNA level detected in TCC samples is respectively 2.3- and 4.1-fold lower than those detected in normal peripheral mononucleocyte and testis samples. In the small number of normal bladder samples analysed ($n=5$), *MRP* gene transcript levels varied over a comparable range and level to those observed in bladder tumours (70% coefficient of

**Figure 3** (**a**) Mean *MRP* mRNA levels for each TCC and normal bladder sample analysed, with each sample represented by a single point. Tumour samples are shown grouped according to stage (▲) and grade (△), and normal bladder samples are shown as stripped urothelium (◇) or whole bladder wall (◆). (**b**) Histograms showing the distribution of individual tumour *MRP* mRNA levels in groups of high-(G3) and low-(G1/2) grade bladder tumours. Tumours are shown grouped according to their level of *MRP* mRNA expression. Population mean and median *MRP* mRNA levels are shown for each group of tumours.

**Figure 4** (**a**) The relationship between pretreatment *MRP* mRNA levels and the response to chemotherapy. Tumour *MRP* mRNA levels (mean *MRP*/18S ratio ± s.e.) are shown grouped according to regimen received and response to chemotherapy (CR, complete response, ▭; PR, partial response, ▨; NR, no response (no change or progressive disease), ■). (**b**) MRP mRNA levels (mean *MRP*/18S ratio ± s.e.) in paired samples taken before (▭) and following (■) chemotherapy from four individual patients, shown grouped according to chemotherapy regimen received. Biopsies were taken from patient 4 at 3 and 6 weeks following the end of treatment. Probability values (by *t*-test or Welch's *t*-test for unequal variances*) for increases/decreases in expression observed following treatment are shown.

**Figure 5** Correlation between the mean tumour *MRP*/18S (*y*-axis) and *MDR1*/18S (*x*-axis) ratios in all samples.

variation). Given the similarity between the range and level of *MRP* mRNA expression in normal bladder and TCC samples, it would be of future interest to determine whether *MRP* mRNA levels found in individual tumours are indeed reflective of those found in the surrounding normal tissue by investigating multiple biopsies from the bladders of individual patients that are commonly taken for prognostic purposes.

Little is currently known regarding the mechanisms of *MRP* gene regulation that underlie the variations in *MRP* mRNA levels observed between individual tumours. For *in vitro* selected MDR cell lines, gene amplification has been demonstrated to account at least partially for increased *MRP* gene expression in the majority of cell lines previously reported, however, in the absence of any chemotherapeutic selection pressure, this seems unlikely in untreated tumours. Zhu and Center (1994) recently cloned the MRP promoter region from HL60/ADR cells, revealing the presence of consensus domains for a number of regulatory elements, including a consensus binding sequence for the c-*fos*/c-*jun*(AP1) transcription factor complex. This suggests that growth signal transduction pathways and their deregulation during oncogenic transformation and tumour progression may play an important role in the transcriptional regulation of the *MRP* gene. Indeed, Bordow *et al.* (1994) recently reported that *MRP* mRNA levels were highly correlated ($P=0.0009$, $n=25$) with mRNA levels of the N-*myc* oncogene in childhood neuroblastoma. Thus, while studies have yet to demonstrate a causal link, observations do suggest that oncogenes and growth factors are likely to influence MRP gene expression.

The association observed between *MRP* mRNA expression and tumour grade may shed some light on the mechanism of *MRP* gene regulation in tumours. Our results show that the proportion of tumours with low *MRP* mRNA levels (*MRP*/18S ratio $<1\times10^{-5}$) is significantly greater in the high-grade compared with the low- and moderate-grade group (55% *vs* 8%). However, it was notable that the presence of a single high MRP-expressing tumour in the high-grade group (see Figure 3a) may mask a more pronounced difference. Because of its markedly high *MRP* mRNA level, the histology of this sample was reassessed. This revealed the tumour to be of mixed nature, with sufficient multiple squamous cell carcinoma (SCC) elements present among TCC regions to suggest its reclassification as borderline SCC. SCC represent <10% of all bladder tumours, and has a worse prognosis than TCC (<30% 5 year survival; Raghavan, 1988). Its SCC nature may potentially explain its atypical *MRP* mRNA level. More interestingly, this sample suggests that *MRP* mRNA levels may be higher in SCC than TCC of the bladder, and may be worthy of further investigation, especially given the worse prognosis of SCC tumours. However, even with the inclusion of this sample, our results demonstrate a trend towards down-regulation of *MRP* mRNA expression in high-grade TCC tumours, and suggest that this may be a general consequence of the loss of tumour differentiation and late-stage progression events associated with high-grade disease.

Varying degrees of lymphocytic infiltration are found in bladder tumours which, given their relatively high MRP expression levels, should be considered for their influence on the tumour measurements. The most extensive data on lymphocytic infiltration of bladder tumours has been published by Lipponen *et al.* (1993) based on 514 patients. Interestingly, this study reports increased lymphocytic infiltration in late-stage high-grade tumours, with 26% of G1/G2 tumours showing moderate or dense infiltration compared with 68% of G3 tumours. This would tend to pull the mean tumour MRP levels up in high-grade tumours, whereas we see a trend down. This in turn would suggest that the reduction in tumour cell MRP levels we have observed may be more marked than apparent from the bulk tumour sample measurements.

The clinicopathological relevance of the reduced *MRP* mRNA levels observed in high-grade tumours is uncertain. Reduced *MRP* mRNA levels may represent a marker of advanced disease in TCC of the bladder, and raises questions about their prognostic significance. The preliminary survival data accumulated to date (28.1 month mean follow-up in surviving patients) suggest that low *MRP* mRNA levels do not identify a subgroup of high-grade tumours with worse prognosis, with equivalent mortality rates observed in subgroups of high-grade tumours with high (*MRP*/18S $>1\times10^{-5}$, $n=6$) and low (*MRP*/18S $<1\times10^{-5}$, $n=6$) *MRP* mRNA levels (four out of six patients dead from their disease in each case). *MRP* mRNA expression did not predict tumour recurrence and progression within the group of superficial bladder tumours studied, however the numbers involved to date ($n=13$) are small, and only two tumours in this group had low *MRP*/18S ratios ($<1\times10^{-5}$). Further clinical studies are therefore required to assess whether low *MRP* mRNA levels predict an adverse prognosis in superficial disease, as well as data on longer term survival follow-up in high-grade tumours.

The pattern of *MRP* mRNA expression observed with respect to tumour grade is markedly different from that which we have previously reported for the *MDR1* gene in untreated bladder tumours, where pooled mean *MDR1* mRNA levels were significantly higher in high-grade than low-grade tumours, and were associated with a poorer patient prognosis (Clifford *et al.*, 1994). Thus, despite an apparent structural and functional similarity, the expression patterns of the *MDR1* and *MRP* genes appear to be markedly different in bladder cancer, and reflect the differences in their pattern of expression observed between different tissues (Cole *et al.*, 1992). The present study demonstrates no correlation between the mRNA levels of the *MDR1* and *MRP* genes and thus provides no evidence to suggest co-regulation or expression of the two genes.

No evidence was found to support a role for high pretreatment *MRP* mRNA levels as a negative determinant of the response of untreated bladder tumours to the CMV or EPICM regimens, based on the limited number of samples investigated. Similarly, no consistent evidence was found to suggest that MRP-mediated drug resistance is selected for during treatment with either the EPICM or CMV regimens. Several reasons may potentially explain the lack of associations observed between *MRP* mRNA levels and response. Firstly, that MRP-mediated drug resistance is not functional at the *MRP* mRNA levels reported and is therefore not relevant to bladder cancer, with other mechanisms of resistance involved in the determination of response. This question could be further and specifically tested *in vitro* using *MRP* gene down-regulation strategies in unselected bladder tumour cell lines. Secondly, that with the regimens used, the MRP-substrate drugs (epirubicin and vinblastine) are not being administered at sufficiently efficacious concentrations, and other drugs contained in the regimen (cisplatin and methotrexate) are responsible for any tumour responses observed. A critical examination of this question would require extension of these studies to a larger population of patients, with chemotherapy regimens involving therapeutic dose monitoring to achieve controlled significant doses of those agents to which MRP can confer resistance. Finally, the timing of post chemotherapy tumour sampling (at 2–3 weeks following therapy) may not have allowed sufficient time for tumour repopulation by any chemotherapy-selected resistant cell subpopulations.

The wide variation in *MRP* mRNA levels (190-fold) observed in the high-grade, invasive group of bladder tumours that are routinely treated by chemotherapy remains potentially very important in terms of their differential chemosensitivity. Studies with *MRP* transfectants and *in vitro* selected cell lines (e.g. Grant *et al.*, 1994; Cole *et al.*, 1992; Zaman *et al.*, 1993; Slovak *et al.*, 1993) have shown that far smaller increases (approximately 10- to 100-fold) in *MRP* mRNA levels are associated with or confer marked increases in resistance in MDR cells. However, the ranges over which these variations occur may be different to those observed in bladder tumours. Some useful initial evidence in this regard comes from a recent study by Hasegawa *et al.*

(1995), who reported the presence of detectable levels of the MRP protein (by Western blot) in the unselected TCC cell line, KK47. Using our PCR-based gene transcript assay, we have independently (Clifford, 1994) demonstrated this cell line to have an *MRP*/18S ratio of 8.2 ($\pm$ 2.7) $\times 10^{-5}$ (mean $\pm$ s.e.), which lies within the range of *MRP* mRNA levels presently reported in untreated bladder tumours, thus suggesting that the *MRP* mRNA levels observed in bladder tumours may indeed encode significant levels of the MRP protein. Further studies are required to determine whether the variations in *MRP* mRNA and protein levels observed in bladder tumours occur over a functionally significant range.

In summary, this study reports a pilot investigation demonstrating extensive variation in the levels of *MRP* mRNA expression in TCC of the bladder, with a trend towards low levels to be found more frequently in high-grade invasive TCC of the urinary bladder. However, despite encouraging *in vitro* and initial clinical findings, both the role of the *MRP* gene in clinical drug resistance and its role in the normal, non-neoplastic urothelium (and the consequence of variations in expression) still remain to be defined. The recent development of discriminatory anti-MRP monoclonal antibodies (Flens *et al*., 1994; Hipfner *et al*., 1994) should aid the progress of investigations regarding the relationship between *MRP* mRNA, protein levels/localisation and drug resistance in both tumour and cell line samples, and help to elucidate their significance in terms of tumour response to chemotherapy.

**Acknowledgements**

All clinical procedures described in this study were performed at the Freeman Hospital, Newcastle-upon-Tyne by Mr DJ Thomas (Registrar in Urology) with histological and pathological examinations carried out by the Department of Pathology. GLC4 and GLC4/ADR cell lines were gifts from Dr Coby Meijer, University Hospital, Groningen. This work was funded by the North of England Cancer Research Campaign.

### References

ALMQUIST KC, LOE DW, HIPFNER DR, MACKIE JE, COLE SPC AND DEELEY RG (1995). Characterisation of the Mr 190,000 multidrug resistance protein (MRP) in drug-selected and transfected human tumour cells. *Cancer Res*., **55**, 102–110.

BARRAND MA, HEPPELL-PARTON AC, WRIGHT KA, RABBITTS PH AND TWENTYMAN PR. (1994). A 190-kilodalton protein overexpressed in non-p-glycoprotein-containing multidrug-resistant cells and its relationship to the MRP gene. *J. Natl Cancer Inst*., **86**, 110–117.

BORDOW SB, HABER M, MADAFIGLIO J, CHEUNG B, MARSHALL GM AND NORRIS MD. (1994). Expression of the multidrug resistance-associated protein (MRP) gene correlates with amplification and overexpression of the n-myc oncogene in childhood neuroblastoma. *Cancer Res*., **54**, 5036–5040.

BURGER H, NOOTER K, SONNEVELD P, VAN WINGERDEN KE, ZAMAN GJR AND STOTER G. (1994). High expression of the multidrug resistance-associated protein (MRP) in chronic and prolymphocytic leukaemia. *Br. J. Haematol*., **88**, 348–356.

CLIFFORD SC. (1994). Determinants of chemosensitivity in primary human bladder cancer. PhD Thesis, University of Newcastle upon Tyne.

CLIFFORD SC, THOMAS DJ, NEAL DE AND LUNEC J. (1994). Increased MRD1 gene transcript levels in high-grade carcinoma of the bladder determined by quantitative PCR-based assay. *Br. J. Cancer*, **69**, 680–686.

COLE SPC, BHARDWAJ G, GERLACH JH, MACKIE JE, GRANT, CE, ALMQUIST KE, STEWART AJ, KURZ EU, DUNCAN AMV AND DEELEY RG. (1992). Overexpression of a transporter gene in a multidrug-resistant human lung cancer cell line. *Science*, **258**, 1650–1654.

COLE SPC, SPARKS KE, FRASER K, LOE DW, GRANT CE, WILSON GM AND DEELEY RG. (1994). Pharmacological characterisation of multidrug resistant MRP-transfected human tumour cells. *Cancer Res*., **54**, 5902–5910.

DAVIES JM. (1992). Occupational and environmental factors in bladder cancer. In *Scientific Foundations of Urology*, 2nd edn, Chisholm GD and Innes-William D (eds), pp. 723–727. Heinemann: London.

ENDICOTT JA AND LING V. (1989). The biochemistry of P-glycoprotein mediated resistance. *Annu. Rev. Biochem*., **58**, 136–171.

FLENS MJ, IZQUIERDO MA, SCHEFFER GL, FRITZ JM, MEIJER CJLM, SCHEPER RJ AND ZAMAN GJR. (1994). Immunochemical detection of the multidrug resistance-associated protein MRP in human multidrug-resistant tumour cells by monoclonal antibodies. *Cancer Res*., **54**, 4557–4563.

GONZALEZ IL AND SCHMICKEL RD. (1986). The human 18S ribosomal RNA gene: evolution and stability. *Am. J. Hum. Genet*., **38**, 419–427.

GRANT CE, VALDIMARSSON G, HIPFNER DR, ALMQUIST KC, COLE SPC AND DEELEY RG. (1994). Overexpression of multidrug resistance-associated protein (MRP) increases resistance to natural product drugs. *Cancer Res*., **54**, 357–361.

HARKER WG, MEYERS FJ, FREIHA, FS, PALMER JM, SHORTLIFFE LD, HANNIGAN JF, MCWHIRTER KM AND TORTI FM. (1985). Cisplatin, methotrexate and vinblastine (CMV): an effective chemotherapy reigmen for metastatic transitional cell carcinoma of the urinary tract. A Northern California Oncology Group study. *J. Clin. Oncol*., **3**, 1463–1470.

HASEGAWA S, ABE T, NAITO S, KOTOH S, KUMAZAWA J, HIPFNER DR, DEELEY DR, COLE SPC AND KUWANO M. (1995). Expression of multidrug-associated protein (MRP), MDR1 and DNA topoisomerase II in human multidrug-resistant bladder cancer cell lines. *Br. J. Cancer*, **71**, 907–913.

HIPFNER DR, GAULDIE SD, DEELEY RG AND COLE SPC. (1994). Detection of the Mr 190,000 multidrug resistance protein, MRP, with monoclonal antibodies. *Cancer Res*., **54**, 5788–5792.

LIPPONEN PK, ESKELINEN HJ, JAUHIAINEN K, HARJU E AND TERHO R. (1993). Tumour infiltrating lymphocytes as an independent prognostic factor in transitional cell bladder cancer. *Eur. J. Cancer*, **29**A, 1757–1761.

LOWE T, SHREFKIN J, YANG SQ AND DIFFENBACH CW. (1990). A computer program for selection of oligonucleotide primers for polymerase chain reactions. *Nucl. Acid Res*., **18**, 1757–1761.

MARSH W, SICHERI D AND CENTER MS. (1986). Isolation and characterisation of adriamycin resistant HL60 cells which are not defective in the initial intracellular accumulation of drug. *Cancer Res*., **46**, 4053–4057.

MOSTOFI FK, DAVIS CJ AND SESTERHENN IA. (1988). Pathology of tumours of the urinary tract. In *Diagnosis and Management of Genitourinary Cancer*. Skinner DG and Lieskovsky G (eds) pp. 83–117. WB Saunders: London.

MULLER M, MEIJER C, ZAMAN GJR, BORST P, SCEPER RJ, MULDER NH, DE VRIES EGE AND JANSEN PLM. (1994). Overexpression of the gene encoding the multidrug resistance-associated protein results in increased ATP-dependent glutathione s-conjugate transport. *Proc. Natl Acad. Sci. USA*, **91**, 13033–13037.

NOONAN KE, BECK C, HOLZMAYER TA, CHIN JE, WUNDER JS, ANDRULIS IL, GAZDAR AF, WILLMAN CL, GRIFFITH B, VON HOFF DD AND RONINSON IB. (1990). Quantitative analysis of MDR1 gene expression in human tumors by polymerase chain reaction. *Proc. Natl Acad. Sci. USA*, **87**, 7160–7164.

RAGHAVAN D. (1988). The management of bladder cancer: current practice and future prospects. In *Management of Bladder Cancer*. Raghaven D (ed.) pp. 317–332. EJ Arnold: London.

SCHNEIDER E, COWAN KH, BADER H, TOOMEY S, SCHWARTZ GN, KARP JE, BURKE PJ AND KAUFMANN SH. (1995). Increased expression of the mutlidrug resistance-associated gene in relapsed acute leukaemia. *Blood*, **85**, 186–193.

SLOVAK ML, PELKEY HO J, BHARDWAJ G, KURZ EU, DEELEY RG AND COLE SPC. (1993). Localisation of a novel multidrug resistance associated gene in the HT1080/DR4 and H69AR human tumor cell lines. *Cancer Res*., **53**, 3221–3225.

STERNBERG CN, YAGODA A, SCHER HI, WATSON RC, HERR HW, MORSE MJ, SOGANI PC, VAUGHAN ED, BANDER N, WEISELBERG LR, GELLER N, HOLLANDER PS, LIPPERMAN R, FAIR WR AND WHITMORE WF. (1988). MVAC (methotrexate, vinblastine, doxorubicin, cisplatin) for advanced transitional cell carcinoma of the urothelium. *J. Urol.*, **137,** 663–666.

UICC. (1978). *TNM Classification of Malignant Tumors*, Third edition. International Union against Cancer: Geneva.

ZAMAN GJR, VERSTANTVOORT CHM, SMIT JJM, EIJDEMS EWHM, DE HAAS M, SMITH AJ, BROXTERMAN HJ, MULDER NH, DE VRIES EGE, BAAS F AND BORST P. (1993). Analysis of the expression of *MRP*, the gene for a new putative transmembrane drug transporter, in human multidrug resistant lung cancer cell lines. *Cancer Res.*, **53,** 1747–1750.

ZHU Q AND CENTER MS. (1994). Cloning and sequence analysis of the promoter region of the MRP gene of HL60 cells isolated for resistance to doxorubicin. *Cancer Res.*, **54,** 4488–4492.

ZIJLSTRA JG, DE VRIES EGE AND MULDER NH. (1987). Multifactorial drug resistance in an adriamycin-resistant human small cell lung carcinoma cell line. *Cancer Res.* **47,** 1780–1784.